# Circles within circles: commentary on Ghosal et al. (2013) “Circ2Traits: a comprehensive database for circular RNA potentially associated with disease and traits”

**DOI:** 10.3389/fgene.2014.00459

**Published:** 2015-01-07

**Authors:** John M. Hancock

**Affiliations:** Department of Physiology, Development and Neuroscience, University of CambridgeCambridge, UK

**Keywords:** circular RNAs, MicroRNAs, Databases, Genetic, Parkinson disease, cancer

In the mid-1980s I remember having the (self-appointed) task of running that most user-friendly of molecular biology tools, the denaturing formaldehyde RNA gel, on whole Drosophila embryo extracts (data sadly unpublished). I was struck at the time by the large amount of RNA I detected, and the wide size range (I was particularly interested in ribosomal RNA at the time so the small stuff didn't interest me much; indeed I presumed it was the result of my poor handling of the sample and resultant degradation, no doubt partly true). Thirty years on we understand massively more about the RNA complement of the cell, with new RNA components being discovered at frequent intervals.

One of the most prominent of these “new” RNA classes is the microRNAs (miRNAs), which play a key role in gene regulation by binding mRNAs and inducing their cleavage, destablilsation through shortening of their poly(A) tail or blockage of translation. The miRNAs are an excellent example of the new, more complex view we now have of gene regulation, which supplants the traditional view of gene regulation acting primarily at the DNA level. With this in mind, it should have come as no surprise to learn, as we have recently, that miRNAs themselves also undergo complex patterns of regulation, some of them mediated by other RNA species.

The idea that there may be circular RNAs in cells go back at least as far as the mid-1970s when (Sanger et al., 1976) identified viroids as circular, single-stranded RNAa. Viroids were, however considered to be special cases and although there were some reports of non-viroid circular RNAs in cells (Nigro et al., 1991; Cocquerelle et al., 1992; Saad et al., 1992; Capel et al., 1993; Sinclair and Guarente, 1997), including an electron microscopic description of circular RNAs in cytoplasm (Hsu and Coca-Prados, 1979) there was no systematic evidence for the existence of, or a major role for, circular RNA molecules in cells until the advent of high-throughput sequencing technologies.

Salzman et al. (2012) published an RNA-Seq study of cellular RNA that identified large numbers of circularly permuted exonic sequences. The evidence that many of these transcripts were circular came from their resistance to digestion by RNase R, which degrades linear RNA. The RNAs were not polyadenylated and were enriched in the cytoplasm. Jeck et al. (2013) subsequently detected more than 25,000 distinct circular RNAs in human fibroblasts. Memczak et al. (2013) carried out an in-depth systematic computational analysis of human, mouse and nematode circRNAs, identifying 2000 in human, 1900 in mouse and 700 in nematode. Interestingly, although the bulk of these circRNAs contained an exonic component, a significant fraction were antisense, purely intronic, from UTRs or ncRNAs, or of uncharacterized intergenic origin.

In parallel to these high-throughput observations, Hansen et al. (2011, 2013a,b) identified a role for circular antisense RNAs in regulating the CDR1 genes and interacting with miR-671 and more broadly identified a role for circRNAs as miRNA “sponges,” binding miRNAs and, potentially, releasing them in response to specific regulatory signals (Hansen et al., 2013a). They also identified a possible role of cirRNAs in cancer due to the involvement of mir-7, which has a cognate circRNA crIR-7 (Hansen et al., 2013b).

These observations inevitably lead to an interest in the potential role of circRNAs in human disease more widely. Ghosal et al. (2013) address this by developing a dataset intended to identify possible associations of circRNAs with diseases (and, through inclusion of GWAS SNPs, other traits such as height). Their resource, Circ2Traits, integrates data and predictions from a number of other sources. The basic dataset is Memczak et al.'s (2013) identified circRNAs and their predicted miRNA interactions. Ghosal et al. then compiled interactomic data by identifying miRNA-disease associations, miRNA-mRNA interactions and miRNA-ncRNA interactions predicted and extracted from a number of resources.

An understandable concern with such a resource, which relies heavily on combining *in silico* predictions to come up with additional, secondary predictions is that it may be prone to high levels of false positives and false negatives. The statistic used to test for the likelihood of the circRNA to be enriched for interaction with miRNAs associated with a disease, although Bonferroni corrected (which might of itself introduce false negatives) does not take into account any uncertainty in the underlying predictions. Future developments of this resource will likely need to use a more sophisticated statistical approach. This is not to suggest that Circ2Traits has no utility, however. As an example of its utility, the authors present evidence that their approach identifies a potentially important relationship between CDR1as, which is posited to bind miR-7, and Parkinson's disease. This underlines the value of this resource as a hypothesis-generating tool.

We are at the earliest stages of understanding any role of circRNAs in disease, and in biology more widely. Circ2Traits is likely the first of a number of attempts to bring together evidence on circRNAs that will facilitate experimental analysis of these RNAs.

## Conflict of interest statement

The author declares that the research was conducted in the absence of any commercial or financial relationships that could be construed as a potential conflict of interest.
